# The Use of Ellagic Acid and Annona Muricata Improves Semen Quality in Men with High-Risk Papillomavirus Infection

**DOI:** 10.3390/jcm11164691

**Published:** 2022-08-11

**Authors:** Sandro La Vignera, Livia Basile, Antonio Aversa, Aldo E. Calogero, Agata Grillo, Rossella Cannarella, Laura M. Mongioì, Rosita A. Condorelli

**Affiliations:** 1 Department of Clinical and Experimental Medicine, University of Catania, 95123 Catania, Italy; 2 Department of Drug and Health Sciences, University of Catania, 95123 Catania, Italy; 3 Department of Experimental and Clinical Medicine, University Magna Graecia of Catanzaro, 88100 Catanzaro, Italy; 4 Labogen (Specialized Human Genetics Laboratory), 95124 Catania, Italy; 5 Glickman Urological & Kidney Institute, Cleveland Clinic Foundation, Cleveland, OH 10681, USA

**Keywords:** HPV, male infertility, ellagic acid

## Abstract

**Background:** Few data are currently available on the treatment of patients with HPV infection. In particular, there is no agreement on the use of antioxidants in these patients. Ellagic acid and annona muricata appear to improve HPV clearance in infected women. However, it is presently unknown whether they could enhance the clearance of HPV infection in infertile male patients. **Aim:** To evaluate the effects of a commercially available combined compound containing ellagic acid and annona muricata on semen quality in patients with documented papillomavirus (HPV) infection, and on the frequency of HPV DNA detection in seminal fluid after treatment. In addition, anti-sperm antibodies and the percentage of spermatozoa with fragmented DNA were evaluated. **Materials and methods:** This was a retrospective case-control study including patients attending our center for infertility. Fifty selected patients who were positive for high risk (HR)-HPV with available semen analysis results were consecutively enrolled. Patients were classified into two groups, according to the clinician’s decision to either administer ellagic acid 100 mg and annona muricata 100 mg (combined tablet formulation) for a period of three months (Group A; 25 patients), or to re-evaluate HPV DNA after a period of active surveillance only (protected sexual intercourse) (Group B; 25 patients). **Results:** Group A patients had a mean age of 31.0  ±  11.0 years, while Group B was 33.0  ±  8.0 years old (*p*  > 0.05). After three months of treatment with ellagic acid and annona muricata, all conventional seminal parameters improved more significantly in Group A than in Group B patients: sperm concentration = 45 mil/mL vs. 20 mil/mL (*p* < 0.05); sperm progressive motility = 45% vs. 18% (*p* < 0.05); and normal sperm morphology = 18% vs. 6% (*p* < 0.05). After the treatment, the frequency of persistence of HPV DNA in the seminal fluid was significantly lower in Group A patients compared to those in Group B (12/25 = 48% vs. 22/25 = 88%; *p* < 0.05). Finally, after 3 months, Group A showed a significant reduction in anti-sperm antibodies and in the percentage of spermatozoa with fragmented DNA. **Conclusion:** The results of this study demonstrate, for the first time, the effects of a commercially available combined compound containing ellagic acid and annona muricata on semen quality in patients with HR-HPV infection, and that this therapy is also associated with a significant reduction in the persistence of HPV DNA in the seminal fluid.

## 1. Introduction

Papillomavirus (HPV) infection has been observed with high clinical frequency in infertile patients for many years now [1]. The epidemiological aspects, the transmission risk factors, and the consequences on the quality of seminal parameters (in particular, the high frequency of asthenozoospermia) are very well documented [2]. The mechanism of adhesion of HPV to spermatozoa has been reported, and its effects on non-conventional sperm parameters (e.g., sperm DNA fragmentation, SDF) have been evaluated [3]. Researchers have also documented the ultrasonography characteristics of the male accessory sex glands during HPV infection [4], the different impact on seminal quality in low oncogenic risk genotypes compared to those with high oncogenic risk [5], and the mechanisms favoring abortion after fertilization [6].

Limited data are currently available on the treatment of patients with HPV infection. Vaccines are the first choice to promote an antibody response and achieve a faster clearance of the infection [7]. However, it must be taken into account that the frequency of spontaneous resolution of HPV infections in men is relatively high, especially for asymptomatic forms [7].

Viral and bacterial urogenital infections are known to negatively impact the oxidative balance, thus worsening seminal parameters [8]. Clinicians managing infertile patients with a first HPV-DNA detection in the seminal fluid often recommend a re-evaluation after a few months, mainly just instructing patients to abstain from unprotected sexual intercourse [9]. However, there is no unanimous agreement on this point. While vaccination is generally suggested in the case of the persistence of the infection, the real role of antioxidant therapy in these patients is poorly understood. An antioxidant is a substance that neutralizes or protects cells against the effects of oxidation and free radicals. The antioxidant system includes enzymatic or non-enzymatic factors. Enzymatic antioxidants include superoxide dismutase, catalase, glutathione peroxidase, and glutathione reductase. Many non-enzymatic antioxidants exist, and these include glutathione, cysteine n-acetylcysteine, carotenoids, vitamin C, vitamin E, carnitine, ferritin, L-arginine, transferrin, coenzyme Q10, myo-inositol, lycopene, selenium, zinc, and folate, to name a few [10,11]. The mechanisms of action of these antioxidants include free radical scavenging and neutralization, as well as preserving sperm DNA integrity and mitochondrial function [12]. The use of antioxidants has been suggested in patients with male infertility. The 2019 Cochrane review, which updated the previous 2014 version, concluded that pregnancy rates improve with antioxidants (OR 2.97, 95% CI 1.91–4.63) [11]. Furthermore, antioxidants appear particularly useful in patients with urogenital infections following the eradication of pathogens [13,14].

Ellagic acid is a phenolic compound with antioxidant, chemopreventive, and antiviral activities. Annona muricata is a type of fruit tree with anticancer and antioxidant activities [15,16,17,18,19,20]. Emerging evidence indicates that these compounds promote the clearance of HPV infection in women [21]. However, it is currently unknown whether these molecules enhance the clearance of HPV infection in affected male patients.

Therefore, this study aimed to evaluate the effects of a three-month treatment regime using ellagic acid (100 mg) and Annona muricata (100 mg) on both semen quality and frequency of HPV DNA detection in patients with high-risk (HR) oncogenic HPV infection. Finally, we evaluated the percentage of patients with anti-sperm antibodies, and the percentage of spermatozoa with fragmented DNA, before and after therapy.

## 2. Patients and Methods

### 2.1. Patient Selection

This was a retrospective case-control study performed using the clinical charts of men who consulted the Division of Endocrinology, Metabolic Diseases and Nutrition, at the University of Catania, for infertility.

We consecutively screened the clinical charts of patients attending the outpatient clinic from January 2018 to April 2021. We selected the first 50 patients who were positive for HR-HPV and had undergone semen analysis at the time of the infection.

Exclusion criteria were as follows: the presence of non-HPV-related male accessory gland infection (MAGI), severe oligozoospermia (sperm concentration < 5 mil/mL), any endocrine disease including any type of diabetes mellitus, cigarette smoking, being overweight/obese, mean testicular volume < 12 mL (measured using the Prader’s orchidometer), and any drug use. In particular, to exclude the presence of bacterial MAGI, symptomatic patients and those with certain signs at the physical examination (tenderness of the epididymis or vas deferens, or abnormal rectal exploration) underwent sperm and urethral swab cultures. Patients with positive cultures or with signs/symptoms strongly suggestive of bacterial infection were excluded.

Patients were classified into two groups according to the clinician’s decision at the time of their visits on whether to administer ellagic acid (100 mg) and annona muricata (100 mg) or not. The two molecules were given in a single tablet formulation for three months (Group A; 25 patients), or the patient was re-evaluated for HPV DNA presence after three months of active surveillance only (protected sexual intercourse) (Group B; 25 patients).

### 2.2. Semen Analysis

The clinical charts contained information on sperm parameters that were analyzed at the Unit of Endocrinology, Metabolic Diseases and Nutrition, University of Catania. For each patient and control, semen samples were collected by masturbation into a sterile container after 2–7 d of sexual abstinence, and were analyzed immediately after liquefaction. Each sample was evaluated for seminal volume, pH, sperm count, progressive motility, morphology, and round cell concentration according to the 2010 WHO guidelines [22]. The evaluation of anti-sperm antibodies was carried out using the MAR (Mixed Antiglobulin Reaction) test as described in the manual.

### 2.3. Sperm DNA Fragmentation

SDF was evaluated by flow cytometry using an EPICS XL (Becker Coulter, Milan, Italy). DNA fragmentation was evaluated by terminal deoxynucleotidyl transferase-mediated deoxyuridine triphosphate nick-end labeling (TUNEL) staining. The negative control was obtained through the non-addition of terminal deoxynucleotidyl transferase to the reaction mix, while the positive control was obtained by pre-treating spermatozoa with 1 mg/mL of RNase-free deoxyribonuclease I (Sigma Chemical, St. Louis, MO, USA) at 37 °C for 60 min before labeling.

### 2.4. Screening of HPV DNA

According to the clinical records, each enrolled patient underwent HPV DNA testing using the procedure described below. The semen sample underwent DNA extraction using PureLink^®^ Genomic DNA Kits (Invitrogen, Carlsbad, CA, USA, Catalog Numbers K1821-04). Real-time PCR (RT-PCT) was used for DNA amplification, using specific primer pairs for the amplification of the L1 region of the viral genome, which is particularly preserved in the different HPV genotypes (f HPV typing, Multiplex Fluorescent-PCR Kit for Human Papilloma Virus (HPV) Genotyping, Experteam s.r.l., Venezia, Italy). As an internal amplification control, the human gene thiosulfate sulfurtransferase mapping to the region 22q13.1 was simultaneously amplified.

To identify high-risk HPV genotypes (HPV 16, HPV 18, HPV 31, and HPV 45), fluorescent PCR Multiplex using fluorescent primer pairs for the target amplification region (E6 and E7), specific for each different HPV viral type searched, was employed. Human polymorphic system short tandem repeats (STRs) served as internal controls and were amplified simultaneously with the viral genotypes. The visualization of the amplified products was performed by means of fluorescent capillary electrophoresis (Genetic Analyzer ABI Prism 3130 with Software Data Collection, Waltham, MA USA). The Standard Gene Scan 500 LIZ (Waltham, MA USA) was used for the detection of the amplified products.

### 2.5. Statistical Analysis

The results are reported as mean  ±  SD throughout the study. The normal distribution of the variables was evaluated using the Shapiro–Wilk test. The Student *t*-test or the Mann–Whitney U-test was applied according to the normal or non-normal distribution of the data, respectively. The statistical analysis was performed using MedCalc Software Ltd. (Ostend, Belgium), version 19.6–64 bit. A *p*-value lower than 0.05 was accepted as statistically significant.

Since most of the data support the concept that sperm motility is the parameter most likely to be affected by HPV infection, we chose this parameter for power calculations. A 32% sperm progressive motility was assumed for the control, based on the WHO 2010 manual. An absolute reduction of 25% was viewed to be of sufficient clinical significance to change practice. The study required a total of 34 participants (17 per group) to preserve 90% power in order to detect an absolute difference of 5% in a two-sided test, taking into account a dropout rate of 5%.

### 2.6. Ethical Approval

This study was conducted at the Division of Endocrinology, Metabolic Diseases and Nutrition, in the university teaching hospital “G. Rodolico-San Marco”, at the University of Catania (Catania, Italy). The protocol was approved by the internal Institutional Review Board, and written informed consent was obtained from each participant after a full explanation of the purpose and nature of all procedures used. The study was carried out according to the principles expressed in the Declaration of Helsinki.

## 3. Results

The results of this study are reported in Table 1.

After three months of treatment, the percentage of patients with persistence of HR-HPV in Group A—those who received ellagic acid (100 mg) and annona muricata (100 mg) - was found to be significantly lower than that in Group B—those who did not receive drug treatment. Patients in Group A showed a significant increase in sperm concentration, sperm progressive motility, and percentage of spermatozoa with normal morphology, compared with those in Group B. The percentage of patients with anti-sperm antibodies was found to be significantly lower in Group A patients compared with those in Group B. Lastly, the percentage of spermatozoa with fragmented DNA was found to be significantly lower in Group A patients compared with those in Group B.

## 4. Discussion

The results of this study showed that the use of the combination of ellagic acid (100 mg) and annona muricata (100 mg) was associated with an improvement in the semen quality of infertile patients with high-risk oncogenic papillomavirus infection, as well as with an increased clearance of the infection, a reduction in the percentage of patients with anti-sperm antibodies, and a reduction in the percentage of spermatozoa with fragmented DNA.

Ellagic acid is a bioactive plant-based polyphenolic molecule, present in several flowering plants, especially in eudicotyledons [23]. It is considered to be a broad-spectrum polyphenol that finds applications in both the pharmaceutical and cosmetic fields, mainly because of its potent antioxidant activity [24]. Its chemical structure consists of a dilactone of hexahydroxydiphenic acid (HHDP), a gallic acid derivative, which results from the union of two diphenol rings through two lactone links. Thus, the presence in the molecule of both hydrogen bonding donor (-OH) and hydrogen acceptor (-C=O) groups represent the key factor in the comparison and explication of its antioxidant activity and effective scavenging properties against free radicals and reactive species of oxygen (ROS). Interestingly, microflora metabolites of ellagic acid, urolithins, also have antioxidant activity [25].

At physiological pH, ellagic acid interacts with several species of free radicals, and this is an important property, given the prevalence of these molecules in the biological environment. In addition, it can be considered to be an uncommon and versatile antioxidant, because it is continuously reproduced after deactivating two free radicals per cycle, providing a “free radical scavenging cascade”. Thus, ellagic acid ensures continuous and high protective effects against oxidative stress, even at low concentrations [26].

Alongside ellagic acid’s antioxidant effects, several authors have suggested other mechanisms by which ellagic acid interferes with biological processes under conditions of oxidative stress. These include metal chelation [27], binding with DNA [28], increased antioxidant enzyme activity (involving glutathione peroxidase, glutathione reductase, superoxide dismutase, and catalase) [29], and inhibition of lipid peroxidation [30]. Accordingly, the positive effects of ellagic acid against oxidative stress have encouraged scientists to thoroughly investigate its therapeutic potential for the treatment of various human diseases, such as diabetes [31], cancer [32], and cardiovascular [33] diseases.

The evidence that ellagic acid is active against oxidative stress has mostly been demonstrated through in vitro and in vivo studies [34]. Most clinical research reporting the therapeutic potential of ellagic acid is based on primary ellagic acid sources, and only a few clinical studies have investigated the activity of this supplement alone. Specifically, the effects of ellagic acid on oxidative stress and insulin resistance were investigated in a double-blinded randomized controlled trial (RCT) carried out on 44 patients with diabetes. The results demonstrated that an 8-week administration of ellagic acid (180 mg/day) reduces the levels of blood sugar, blood lipids, and insulin resistance [35]. In a double-blinded RCT including 60 women with PCOS, daily administration of ellagic acid (200 mg) for 8 weeks improved the status of oxidative stress and inflammatory asset, which reduced the complications of the diseases [36]. Several molecular mechanisms are considered to explain the beneficial effects of ellagic acid. These include its ability to efficiently hinder the increasing ROS levels produced by glycation reactions and formation of advanced glycation end products [37], as well as the stimulation of the activity of antioxidant enzymes such as glutathione peroxidase and superoxide dismutase. In addition, some authors have suggested that polyphenols might interfere with the activation of peroxisome proliferator-activated receptor gamma transcription factors, thus improving the sensitivity of insulin to its receptors [38]. This might explain the decrease in blood sugar, hemoglobin A1c, insulin, and insulin resistance. Furthermore, ellagic acid has been found to impact cholesterol metabolism, lowering total cholesterol, triglycerides, and low-density lipoprotein levels.

Ellagic acid has been shown to have antitumor effects via the following mechanisms: apoptosis induction, blocking of tumoral proliferation, prevention of viral infection, and interference with inflammation [39]. Experimental evidence has shown antioxidant and proapoptotic activity of ellagic acid in cancer cells exerted by p53/p21 (WAF1/CIP1) expression induced through G1 arrest and activation of gene p21. Accordingly, the effects of ellagic acid were evaluated as a support therapy for chemotherapeutic treatment in men with hormone-refractory prostate cancer [40]. In this study, a significant decrease in systemic toxicity due to chemotherapy was found in the group of patients treated with ellagic acid compared with those receiving chemotherapy alone. Furthermore, positive outcomes in terms of overall survival and objective response were observed, as well as in antigen-specific prostatic reduction, pain control, and quality of life, although these results did not reach statistical significance.

Thus, the cytoprotective, anticytotoxic, and antioxidant effects of ellagic acid contribute to its anticancerogenic action, as demonstrated by an increase in therapy tolerance. Ellagic acid is primarily an antioxidant molecule, and it has been extensively studied for different positive health effects, among which is its ability to modulate the body’s defense system against pathogens such as viruses [41]. HPV belongs to a DNA virus family, and is able to infect basal epithelial cells of the skin or mucosae tissue of the anogenital and upper aero-digestive tract. HPV infection occurs via sexual transmission and represents the main risk determinant for cervical cancer [42]. HPV infection can also cause cancer of the anogenital tract, including the vulva, vagina, anus, and penis [43]. The interplay between carcinogenesis, HPV, and oxidative stress has already been shown [44]. At the basis of this mutual response, two mechanisms are supposed to occur. On the one hand, cervical carcinogenesis can be promoted by the genotoxic activity from the oxidative stress and genomic instability induced by HPV cooperating independently, causing molecular injury in generating neoplastic cells. On the other hand, oxidative stress contributes to the initiation and/or propagation of cancer generated by HPV [21,44].

The therapeutic approaches employed in the treatment of HPV-related cancers, such as surgery, chemo- and radiotherapy are considered insufficient [45]. Evidence from experimental studies elucidating the possible protective effects of ellagic acid are persuasive [46]. Ellagic acid induces apoptosis and inhibits melanoma cell growth in C7BL/6 immunocompetent mice [47]. In human cervical carcinoma Caski cells, ellagic acid stimulated G1 cell cycle arrest and apoptosis [48]. In addition, in HeLa cells, ellagic acid was found to determine the inhibition of the AKT/mTOR signaling pathway via an increased expression of Insulin Like Growth Factor Binding Protein 7 (IGCFBP7). The invasion of HeLa cells and the rate of apoptosis in the presence of ellagic acid were considerably lower than those in the control group [49].

The antiviral and chemoprotective effects of ellagic acid in a group of women with HPV cervical infection were evaluated by Morosetti and colleagues. In this study, the administration of ellagic acid (160 mg daily) for 12 months resulted in a protective effect on cervical cells, whereas no effects on viral clearance and virus integration were found in either the control or treatment groups. Among the mechanisms hypothesized, it seems that apoptosis stimulation, promotion of DNA repair, and inhibition of the cell cycle underlying the antioxidant capacity of ellagic acid can counteract the damage caused by oxidative stress, thus guaranteeing effective maintenance of the body’s natural defense mechanisms, as well as it having an organ-cytoprotective action [21]. A randomized trial by Le Donne and colleagues assessed the impact of ellagic acid and annona muricata in women with low squamous intraepithelial lesion (L-SIL) from high-risk human papilloma virus (HR-HPV). Interestingly, the results showed that women supplemented for 6 months manifested a stronger ability to eliminate HR-HPV compared to the control group. Furthermore, biopsy data revealed that the expression of tumor oncosoppressor p21 in the cervical lesion thickness markedly increases, demonstrating the impact of ellagic acid on the modulation of the tumoral cell cycle [50].

To our knowledge, this is the first evidence regarding the use of phytotherapy in the treatment of male infertility associated with HPV infection with high oncogenic risk. Our research will continue with the study of more numerous case series, and we will later attempt to define further molecular mechanisms that can help to better explain the results obtained.

## Figures and Tables

**Table 1:** Sperm parameters and prevalence of HPV DNA in the semen fluid of patients before and after 3 months of either treatment with ellagic acid (100 mg) and Annona muricata (100 mg) (Group A), or observation only (Group B).

Parameters	Group A		Group B		Reference Values
	Before Treatment	After Treatment	Before Treatment	After Treatment	
Presence of HPV DNA	25/25 (100%)	12/25 (48%) *	25/25 (100%)	22/25 (88%)	/
Sperm concentration (mil/mL)	30.8 ± 13.0	48.2 ± 13.0 *	27.8 ± 6.0	23.2 ± 8.0	>15
Sperm progressive motility (%)	15.2 ± 8.0	32.0 ± 8.0 *	16.4 ± 11.0	14.5 ± 7.0	>32
Sperm normal morphology (%)	11.6 ± 9.0	18.2 ± 10.0 *	12.8 ± 8.0	13.2 ± 6.0	>40
Mixed antiglobulin reaction test (number of positive)	7/25 (28%)	1/25 (4%) *	5/25 (20%)	5/25 (20%)	/
Sperm DNA fragmentation (%)	4.3 ± 0.4	1.6 ± 0.8 *	4.1 ± 1.6	4.4 ± 1.2	<4.6

* *p* < 0.05 vs. Group B after treatment.

## Data Availability

The data presented in this study are available on request from the corresponding author. The data are not publicly available since they belong to an ongoing project.
